# Detection of Circulating Tumor Cells Using Membrane-Based SERS Platform: A New Diagnostic Approach for ‘Liquid Biopsy’

**DOI:** 10.3390/nano9030366

**Published:** 2019-03-05

**Authors:** Agnieszka Kamińska, Tomasz Szymborski, Evelin Witkowska, Ewa Kijeńska-Gawrońska, Wojciech Świeszkowski, Krzysztof Niciński, Joanna Trzcińska-Danielewicz, Agnieszka Girstun

**Affiliations:** 1 Institute of Physical Chemistry, Polish Academy of Sciences, Kasprzaka 44/52, 01-224 Warsaw, Poland; tszymborski@gmail.com (T.S.); ewitkowska@ichf.edu.pl (E.W.); knicinski@ichf.edu.pl (K.N.); 2 Faculty of Materials Science and Engineering, Warsaw University of Technology, Wołoska 141, 02-507 Warsaw, Poland; ewakijenska@gmail.com (E.K.-G.); wojciech.swieszkowski@pw.edu.pl (W.Ś.); 3 Department of Molecular Biology, Institute of Biochemistry, Faculty of Biology, University of Warsaw, Miecznikowa 1, 02-096 Warsaw, Poland; jtd@biol.uw.edu.pl (J.T.-D.); agirstun@biol.uw.edu.pl (A.G.)

**Keywords:** surface-enhanced Raman spectroscopy, circulating tumor cells (CTC), prostate cancer (PC3), cervical carcinoma (HeLa), label-free detection

## Abstract

The detection and monitoring of circulating tumor cells (CTCs) in blood is an important strategy for early cancer evidence, analysis, monitoring of therapeutic response, and optimization of cancer therapy treatments. In this work, tailor-made membranes (MBSP) for surface-enhanced Raman spectroscopy (SERS)-based analysis, which permitted the separation and enrichment of CTCs from blood samples, were developed. A thin layer of SERS-active metals deposited on polymer mat enhanced the Raman signals of CTCs and provided further insight into CTCs molecular and biochemical composition. The SERS spectra of all studied cells—prostate cancer (PC3), cervical carcinoma (HeLa), and leucocytes as an example of healthy (normal) cell—revealed significant differences in both the band positions and/or their relative intensities. The multivariate statistical technique based on principal component analysis (PCA) was applied to identify the most significant differences (marker bands) in SERS data among the analyzed cells and to perform quantitative analysis of SERS data. Based on a developed PCA algorithm, the studied cell types were classified with an accuracy of 95% in 2D PCA to 98% in 3D PCA. These results clearly indicate the diagnostic efficiency for the discrimination between cancer and normal cells. In our approach, we exploited the one-step technology that exceeds most of the multi-stage CTCs analysis methods used and enables simultaneous filtration, enrichment, and identification of the tumor cells from blood specimens.

## 1. Introduction

Circulating tumor cells (CTCs) are shed from the cancerous mass of the primary tumor cells and may get into the peripheral blood and then transfer to distant tissues causing expansion of the cancer in the metastasis form [1]. CTCs are a crucial source of genetic material for clinical analysis, e.g., tumor diagnostics, and selection and monitoring of cancer therapy [2,3,4,5,6]. Tissue biopsies are invasive and expensive, therefore the detection and characterization of tumors based on CTC analysis in a sample of peripheral blood, known as ‘liquid biopsy’, is the subject of special interest. A variety of techniques have been developed for isolation, capturing, and analysis of circulating tumor cells. The most widely used approaches include polymerase chain reaction (PCR) and reverse transcription PCR (RT-PCR) [7], immunofluorescence [8,9,10], fluorescence-based cytometry [11], fluorescence scanning microscopy [12,13,14], and label-free biochemical separation [15]. Recently, the microfluidic devices based on miniaturized nanomaterials and microfluidic reactions improved the sensitivity of detection and enabled continuous single-cell analysis [16,17]. The current ‘gold standard method’ of CTC detection and enumeration is the CellSearch system (Veridex LLC) for the analysis of CTCs in metastatic breast [18], prostate [19], and colon cancer patients [20]. In this method ferromagnetic beads are coated with antibodies against the epithelial cellular adhesion molecule (EpCAM), then immunostained with fluorescently labeled anti-cytokeratin (CK, an epithelial intermediate filament), anti-CD45 (a membrane antigen expressed by leucocytes) antibodies, then stained with DAPI (4′,6-diamidino-2-phenylindole, a nuclear stain), and finally counted by automated cell image capture and analysis even from a 7.5 mL blood sample [21].

However, it should also be highlighted that all the above described techniques require the use of specific molecular markers for CTCs detection and are time-consuming and/or expensive, which limits these methods for routine clinical analysis. For example, RT-PCR enables only the examination of a limited number of genes at the same time and does not permit the morphological analysis of cells in subsequent tests [22]. Moreover, the ability of this technique to detect multiple cancer markers might by hindered by the lack of appropriate tumor marker expression. In all the above mentioned methods, the outcomes have to be confirmed by histopathological examination of the tissue collected during biopsy. In order to reduce high medical costs of biopsy, we attempted to develop a new approach to cancer diagnosis based on the Raman technique.

Surface-enhanced Raman spectroscopy (SERS) is a highly sensitive and specific method that allows for the detection and characterization of various compounds through their capability to generate specific molecular fingerprint signals. SERS spectroscopy reveals the huge enhancement of Raman scattering signals of molecules adsorbed on specially prepared metallic nanostructures, usually made of silver, gold, or copper [23]. The two main mechanisms, electromagnetic (EM) and chemical (CT), can increase Raman peaks intensities by 9–14 orders of magnitude relative to normal Raman spectroscopy, which gives the possibility of single molecule detection [24]. The huge enhancement factor (huge sensitivity), high selectivity, possibility of label-free, rapid, and non-destructive analysis leads to an increase in the practical applications of this technique, especially in biomedical and analytical studies.

Recently, various studies have shown the capability of SERS in tumor cells identification [25,26,27]. Zhang et al. presented a novel strategy based on the nitrocellulose membrane and SERS imaging method, which can be used for both CTC enrichment and detection [28]. Jun et al. [29] developed silica-encapsulated magnetic nanoparticles (MNPs) with unique properties for cancer cell targeting and identification. Wang et al. used SERS-active nanoparticles modified with epidermal growth factor (EGF) peptide as a targeting ligand for efficient CTC detection in blood plasma [30]. Wen et al. successfully developed a method which has an ability to quickly respond and can be used for CTC capturing and detection. The CTC capturing and detection efficiency was also proved via real blood samples from clinical subjects using magnetic nanospheres [31]. Shi et al. [32] reported detection of cervical carcinoma (HeLa) cells using the designed folate-conjugated SERS-active nanoparticles and the magnetic tapping strategy. Krafft et al. [33] presented a microfluidic chip combined with optical tweezers to collect the normal Raman spectra of circulating cells.

Most of the presented SERS-based methods of CTC analysis require an enrichment step of a few CTCs from the blood and an extra labelling strategy for preparation, e.g., Raman reporter or peptides encoded NPs and/or tumor cells pre-labelled with NPs [29], which increases the cost and time of analysis.

Currently, various methods have been explored to optimize the properties of SERS platforms in terms of size, shape, and composition of used plasmonic nanostructures [34,35,36,37]. Even though many efficient SERS supports have been developed [38,39], there are some disadvantages associated with them, e.g., low stability over time, and inability to perform in situ measurements and sample mapping. To avoid these problems, new production strategies of SERS platforms should be developed in order to introduce SERS techniques to standard biomedical and analytical applications. We have demonstrated that polymer mats covered via the PVD (physical vapor deposition) technique with gold or gold–silver alloy may work as a very efficient SERS platform for the identification of bacteria from environmental, food, and human body fluid samples [40,41].

In our approach we offer a simple method that is optimal for CTC isolation, enrichment, detection, and molecular analysis. We have elaborated the procedure of membrane-based SERS platforms (MBSP) with appropriate pore sizes that permit the separation and enrichment of the prostate cancer cell line (PC3), cervical carcinoma cell line (HeLa), and leucocytes—an example of healthy cells in the blood samples. By covering tailor-made SERS substrates with a thin layer of Ag–Au alloy, the high enhancement of Raman signals of all studied CTCs was achieved.

Additionally, to improve the efficiency of discrimination between cancer and blood cells, principal component analysis (PCA) was adopted. PCA is one of the most commonly used methods in the classification of SERS data, which enables the identification of the spectral differences among the studied samples, extracting the characteristic marker bands and biochemical information from SERS spectra. The presented strategy may lead to development of new tools in CTC therapy.

## 2. Experimental Section

The prostate cancer (PC3) and cervical carcinoma (HeLa) cell lines used in this work were obtained from the European Collection of Cell Cultures (ECACC), Sigma-Aldrich (St Louis, MO, USA). PC3 and HeLa cells were cultured in RPMI-1640 and DMEM media, respectively. Both media were supplemented with 10% fetal bovine serum (FBS), streptomycin (100 μg/mL), and penicillin (100 U/mL). The cell cultures were cultivated at 37 °C, in a humidified atmosphere of 5% CO_2_. During experiments the cancer cells were cultured in 25 cm^2^ cell culture flasks. After reaching 80% of confluence, the cells were suspended in phosphate buffer saline (PBS) buffer and trypsinized (0.05% trypsin, 0.02% EDTA solution). Subsequently, the cells were collected, centrifuged at 250 × g for 5 min at room temperature, re-suspended in PBS and centrifuged repeatedly. After the last centrifugation the sample containing 20 μL of PBS was obtained and stored on ice. All the chemical reagents were obtained from Sigma-Aldrich (St. Louis, MO, USA).

During the preparation of prostate cancer (PC3) and cervical carcinoma (HeLa) cell lines for SERS experiments we used concentrations reflecting the population of cancer cells in metastasis. The initial concentration (after the cultivation step) of cancer cells in PBS was 0.44 × 10^6^ cells/mL for PC3 and *ca.* 10^6^ cells/mL for HeLa, respectively, and was further diluted to the final concentration of *ca.* 40 cells in 1mL of blood.

The human blood samples derived from ten healthy volunteers, available courtesy of the Regional Blood Center (Warsaw, Poland), were used in our studies. An informed consent was obtained from all subjects (healthy volunteers). The performance of all experiments was in agreement with the institutional guidelines and relevant laws and approved by the Ethics and Bioethics Committee of Cardinal Stefan Wyszyński University (Warsaw, Poland).

### 2.1. Leucocyte Isolation

Whole blood samples were lysed with five volumes of hypotonic erythrocyte lysis buffer (RBCL, A&A Biotechnology, Gdynia, Poland) from at least 20 mL of peripheral blood. After 15 min of incubation on ice and centrifugation (3000× *g*), the plasma-free leukocytes were re-suspended in PBS solution at a concentration of 2 × 10^7^ cells/mL.

### 2.2. Fabrication of Membrane-Based SERS Platforms (MBSP) via Electrospinning

Poly(l-lactic acid)-co-poly(ε-caprolactone) (P(LLA-CL)) with a ratio of 70:30 used for fiber fabrication was purchased from Evonik (Witten, Germany). Prior to electrospinning, two types of P(LLA-CL) solutions with concentrations of 10% and 14% (w/v) were prepared by dissolving polymer powder (crystals) in 1,1,1,3,3,3-hexafluoro-2-propanol (Fluorochem, Hadfield, Derbyshire, UK) and stirring overnight in ambient conditions. The solutions were then individually placed in 10 mL plastic syringes.

The electrospinning process was carried out under optimized conditions with the use of NANON-01A (MECC Co., Ltd.; Fukuoka, Japan). Two 27 G steel needles were connected with syringes using polytetrafluoroethylene (PTFE) tubes, fixed to the moving head with a constant linear velocity of 100 mm/min, and attached to high voltage of 15 kV provided by a built-in power supply. The distance between the needles was 25 mm, and the tips-to-collector distance was set at 150 mm. The feed rate for both solutions was 1.0 mL/h. The working width of the moving head was 100 mm. The nanofibrous meshes were collected on the aluminum covered steel plate and dried in a vacuum drier for 24 h (25 °C, 50 mb).

### 2.3. Sputtering of the Thin Layer of SERS Active Metal

The PVD device (Leica, EM MED020, Heerbrugg, Switzerland) was applied to sputter 40 nm of Ag:Au alloy directly on the polymer fibers. No adhesion layer, i.e., chromium or titanium, was used between the polymer and the Ag:Au alloy layer. The sputtering conditions were: current of 25 mA and pressure of 10^−2^ mbar.

## 3. Instrumentation

### 3.1. SERS Measurements

Measurements were carried out with a Renishaw inVia Raman system (Wotton-under-Edge, Gloucestershire, UK) equipped diode laser emitting a 785 nm laser line. The light from the lasers was passed through a line filter and focused on a sample mounted on an X–Y–Z translation stage with a 50× microscope objective, NA = 0.25. The beam diameter was approximately 2.5 µm. The laser power at the sample was 1.5 mW. The experiments were performed at ambient conditions using a back-scattering geometry. The spectroscopic maps were acquired by collecting SERS spectra over the previously defined range (36 × 21 μm^2^) at each point on a grid with 3 μm spacing using an automated microscope stage. Typically, 25 SERS spectra for each cell type were acquired. Each spectrum was measured for 30 s.

### 3.2. SEM Measurements

SEM images were acquired from the FEI Nova NanoSEM 450 instrument (Hillsboro, OR, USA) operating at an accelerating voltage of 10 kV and under high vacuum.

### 3.3. Chemometrics—Principal Component Analysis

Principal component analysis (PCA) is a multivariate procedure that can reduce the dimensionality of original raw data to several principal components (PCs). It is an effective technique that gives the possibility to categorize SERS spectra that are readily distinguishable via visual empirical analysis. The calculated PCs contain the most significant information from the whole introduced data set. The PCA was performed using the commercial Unscrambler^®^ software (CAMO software AS, version 10.3, Oslo, Norway). The SERS data of all analyzed cells (leucocytes, HeLa, and PC3 cells) were optimized for PCA using the following steps: (i) smoothing with a Savitzky–Golay filter (Oslo, Norway), (ii) background correction (concave rubber band correction; the number of baseline points was 34 and the number of iterations was 10), and (iii) normalization using OPUS software (Bruker Optic GmbH, 2012 version, Ettlingen, Germany). The PCA was completed based on the NIPLAS algorithm, validation (random with 20 segments), significance 0.05, and a SERS spectra number of 120.

## 4. Results and Discussion

### 4.1. Preparation of the SERS Platform

In this study, we present a novel SERS platform prepared with the use of the electrospinning technique [42] for the label-free analysis of CTCs in blood samples. Fabrication of MBSP consisted of two steps:

(i) Electrospinning of polymer mats with desired parameters, i.e., diameter of the polymer fibers and diameter of the pores;

(ii) Sputtering of thin (usually tens of nanometers) layer of SERS-active metal, e.g., gold, silver, or their alloy via the PVD method.

**Figure 1:**
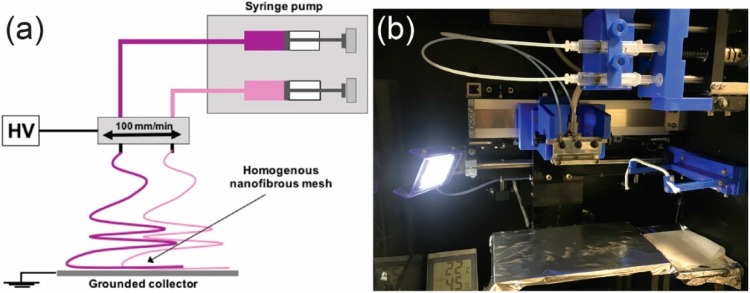
(**a**) Basic layout of the setup utilized for electrospinning, which consists of a high voltage power supply (HV), two syringe pumps, and a grounded collector; (**b**) a photo of experimental setup used in experiments.

The basic scheme of the utilized method for step (i) is shown in Figure 1.

To prepare SERS-active platforms there was a need to coat polymer mats with metal NPs or metal islands. In the case of thin metal islands, it could be done by PVD or vacuum evaporation. In our study the layer of Ag:Au alloy was sputtered on the polymer fibers via the PVD method. The Ag:Au alloy ensured the combination of very high enhancement of the Raman signal provided by Ag with the chemical stability offered by Au [43]. In order to create a platform that provided optimal enhancement of the Raman signal, three different thicknesses of Ag:Au alloys (20, 40, and 80 nm) were tested.

The Ag:Au alloy layer of 20 nm deposited on the polymer mat was too thin to cover the platform and thus to obtain the SERS signal of *p*-MBA (*p*-mercaptobenzoic acid) or tumor cells. As a result, the recorded SERS spectra were derived from the polymer. The polymer mat covered with 40 nm of Ag:Au alloy showed the greatest SERS enhancement. No SERS signals from the polymers were observed. A similar level of enhancement was achieved for the 80 nm Ag:Au layer. Therefore, in the present study, the polymer mats covered with the 40 nm layer were used for all experiments as the most cost-effective. Moreover, the process of sputtering of 40 nm of Ag:Au alloy took only 4 min compared to 8 min for the 80 nm layer.

**Figure 2:**
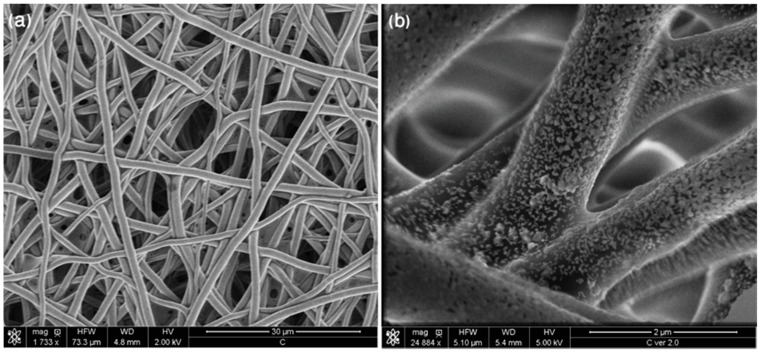
SEM images of electrospun polymer mat coated with gold layer (40 nm) at (**a**) lower and (**b**) higher magnification.

The morphology of the created SERS substrates named Au:Ag/MBSP SERS was examined by scanning electron microscopy (SEM). The SEM images of (P(LLA-CL)) covered with 40 nm of the Ag:Au alloy layer are presented in Figure 2 at smaller and larger magnifications, respectively. As can be seen in Figure 2a, the arrangement of fibers with a diameter of *ca*. 1.5 μm within mats was irregular with the slots between fibers of *ca.* 15 μm that were small enough not to let the tumor cells (with the diameter of 20–28 μm) pass through the (P(LLA-CL)) mat. Additionally, the SEM image shown in Figure 2b reveals that the obtained layer of Ag:Au consisted of semi-spheres with diameters ranging from 40 to 55 nm, and their size was responsible for the enhancement factor of the presented Au:Ag/PBSP SERS substrates, and determined the SERS efficiency of these surfaces.

The SERS platform designed in such a way worked also as a filter, which allowed the separation of circulating prostate cancer (PC3) and cervical carcinoma (HeLa) cells re-suspended in human blood plasma at a concentration of about 40 cells in 1mL of blood.

The main advantage of the proposed method is the fact that it does not require use of separate techniques to perform filtration, enrichment, and examination of tumor cells circulating in blood. Additionally, by combining these three basic steps in detecting cancer cells in one single process, the transfer of the cells from one place/method to another is eliminated. Therefore, the proposed strategy prevents contamination of the samples and disintegration of cell structures, and leads to improvement of the accuracy of analysis and reduction of the time of analysis.

### 4.2. SERS Investigations of Circulating Tumor Cells

**Figure 3:**
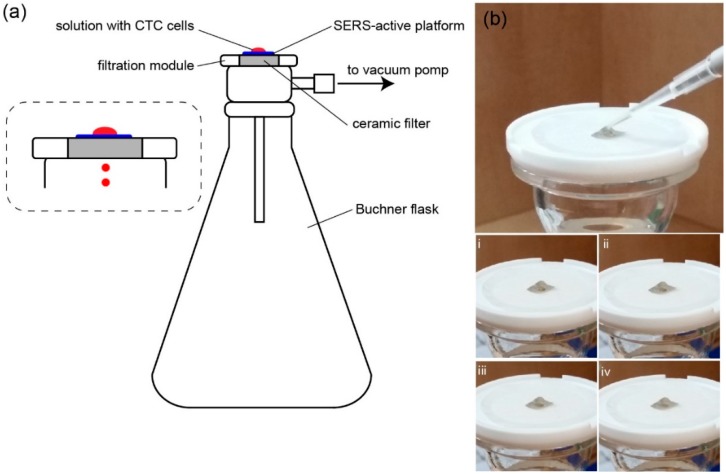
(**a**) The scheme of capturing circulating tumor cells (CTCs) from the blood sample. The system involves: a vacuum pump, Buchner flask, and filter funnel. The surface-enhanced Raman spectroscopy (SERS) platform was placed on the filter funnel and a droplet of blood spiked with CTCs was put on the platform. After turning on the pump, the liquid was sucked through the mat to the flask, whereas the CTCs remained on the surface of the SERS platform. (**b**) Filtration process of the fluid with the CTCs. The setup consists of a ceramic filter and SERS-active platform placed in the very center. After pipetting a small amount of fluid (top) with CTCs, the vacuum pump is turned on and the blood passes through the mat and the ceramic filter to the Büchner flask, whereas the CTCs stay on the SERS-active platform (steps i–iv).

The Au:Ag/MBSP SERS platform worked both as a filter and as an efficient SERS support and allowed for: (i) separation of studied cells from the complex blood sample due to the sizes of MBSP pores (Figure 2) and sizes of particular blood components (Table S1), and (ii) enrichment of circulating tumor cells within a small and defined area of the SERS substrate. Figure 3a illustrates the experimental setup used for the detection of studied cells whereas Figure 3b demonstrates the filtration process.

As the amount of single CTCs in peripheral blood is small [44], the highly-efficient cell enrichment and single cell capturing were essential for further cell examination.

The proposed concept based on spiking blood samples obtained from healthy donors with a known number of HeLa and PC3 cells (40 cells in 1 mL of blood) may have in the future a practical potential in medicine.

In order to push the sample through the device, a constant pressure of approximate 80 × 10^3^ Pa was applied. The whole filtration process took about 4 minutes. Since the pores in the Au:Ag/MBSP SERS platform had a diameter of *ca.* 15 µm and the sizes of blood components and analyzed CTCs did not exceed 15 and 28 µm, respectively (see Table S1, Supplementary Materials), the separation of CTCs from other blood components could be performed. The smaller components of blood plasma passed through the Au:Ag/MBSP SERS platform whilst the largest CTCs remained on the surface of the modified (P(LLA-CL)) mat. As mentioned before, the Ag:Au nanostructures present on the polymer mat fibers are responsible for amplification of the Raman signal of CTCs. Therefore, in the next step the spectroscopic fingerprints of captured CTCs were recorded to perform detailed molecular analysis and identification of studied cells.

In order to collect the reference spectra of all studied cells (PC3, HeLa, and leucocytes as an example of healthy cells) the SERS measurements were performed directly from pre-cultures (see Figure S1, Supplementary Materials). Table 1 presents the main SERS bands observed in analyzed cell spectra and the corresponding bands assignments.

**Figure 4:**
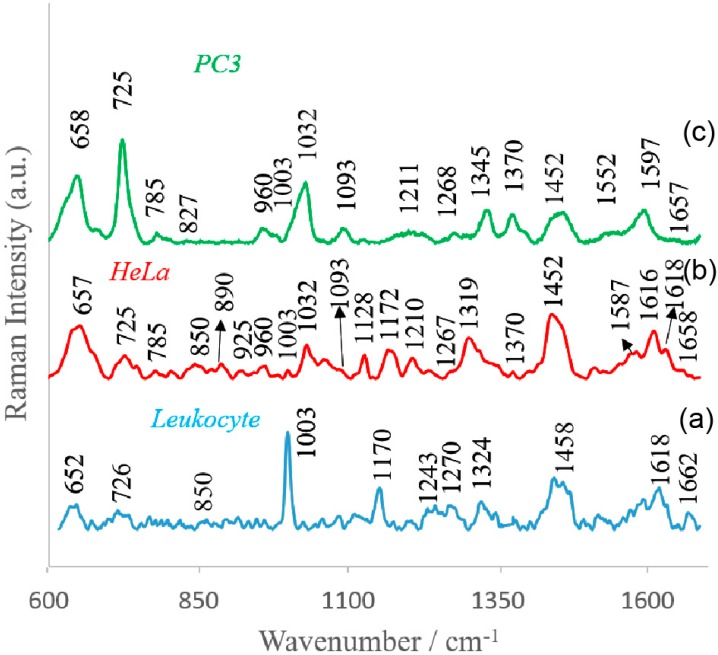
Averaged and normalized SERS spectra of (**a**) leucocytes, (**b**) cervical carcinoma (HeLa), and (**c**) prostate cancer (PC3) cells recorded on polymer-based SERS platform. Experimental conditions: excitation at 785 nm, laser power at 1.5 mW, and 45 seconds integration time. Each SERS spectrum was obtained by averaging at least 25 single spectra from different places on the SERS substrate.

Figure 4 depicts the SERS spectra of the cells isolated from blood samples using the Au:Ag/MBSP SERS platform according to the procedure discussed above. As can be seen these spectra showed differences in the position of some bands and their relative intensities. However, the common bands corresponded to the main components of the eukaryotic cell [45]: nucleic acids, proteins, and lipids were clearly observed in all SERS spectra.

All the spectral fingerprints depicted in Figure 4 corresponded with the reference SERS data in Figure S1. In Figure 4 one can observe that the vibrational modes of nucleic acids were present at 785 and 1093 cm^−1^. The week bands around 1268 and 1660 cm^−1^ were characteristic of amide I and amide III bands, respectively. In all recorded SERS spectra there appeared vibrational modes characteristic of phenylalanine (1003 cm^−1^), tyrosine (850 cm^−1^), and tryptophan (725 cm^−1^). As can be observed, the SERS spectrum of particular cells also had their own specific spectral features. For example, the band at 1345 cm^−1^, which corresponded to adenine and guanine, could be seen in PC3 cells, but not in the HeLa cells and leucocytes. Additionally, the relative intensities of some bands could also be used for differentiation of analyzed cells. To make identification of PC3 and HeLa cells, the ratio of the relative intensities of the bands at 658 cm^−1^/725 cm^−1^ could be used. In the SERS spectra of leucocytes, the most prominent bands appeared at 652 cm^−1^ (C–C twist of tyrosine) [46], 726 cm^−1^ (C–S in protein, CH_2_ rocking, adenine) [47], 1003 cm^−1^ (C–C of phenylalanine) [48], 1170 cm^−1^ (C–H in plane of tyrosine or nucleic acid) [49], 1458 cm^−1^ (nucleic acid nucleotides) [50], and 1618 cm^−1^ (ν(C=C), tryptophan, tyrosine) [51]. All these dissimilarities enabled recognition of circulating cells. The spectroscopic data revealed that the healthy leucocyte cells could be distinguished from tumor cells using bands at 1032 cm^−1^ (CH_2_CH_3_ bending modes of lipids) [51] and 1452 cm^−1^ (structural protein modes of tumors) [52]. The intensive band at 1452 cm^−1^ was assigned to overlapping asymmetric CH_2_ bending and CH_2_ scissors vibrations. The bands of phospholipids, elastin, and collagen were also identified in this region [41]. These differences reflected the changes in biochemical pattern of cancer cells (compared to healthy cells) as the result of carcinogenesis. Table 1 depicts all observed SERS bands with their assignments.

The reproducibility of recorded SERS signals is a crucial parameter, especially in the terms of real clinical applications. We calculated the reproducibility of the SERS signals of leucocyte, PC3, and HeLa cells (usually 40 SERS spectra for each type of cells measured on the same SERS substrate were considered). The calculated standard deviations (RSDs) were performed for the most prominent bands at 725, 1003, and 1035 cm^−1^. The achieved results were 6.2%, 8%, and 7.5%, respectively (Table S2, Supplementary Materials). Additionally, Figure S2 shows examples of representative SERS spectra of all studied cells collected from different points within the same Au:Ag/MBSP SERS platform. However, the changes in relative intensities of the same bands were observed and were related to the effect of molecular orientation in relation to the polarization of plasmon excitations in the metal substrate.

**Figure 5:**
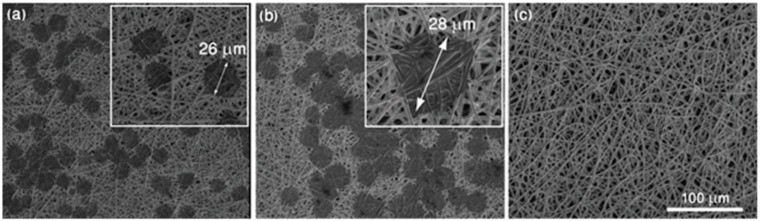
SEM images of SERS platforms after filtration of (**a**) HeLa cells, (**b**) prostate cancer PC3 cells, and (**c**) leucocyte cells. White blood cells were not captured on the polymer based SERS-active platform due to their size, which was smaller than the diameter of pores.

The SEM images (Figure 5) show different types of cells filtered from blood and immobilized on the SERS-active platforms.

It is evident that HeLa and PC3 cells of the size of *ca.* 26 and 28 µm, respectively, could be easily detected on the SERS platform. The leucocytes, which had smaller sizes, passed through the pores and could not be identified by SEM imaging.

### 4.3. Principal Component Analysis

**Figure 6:**
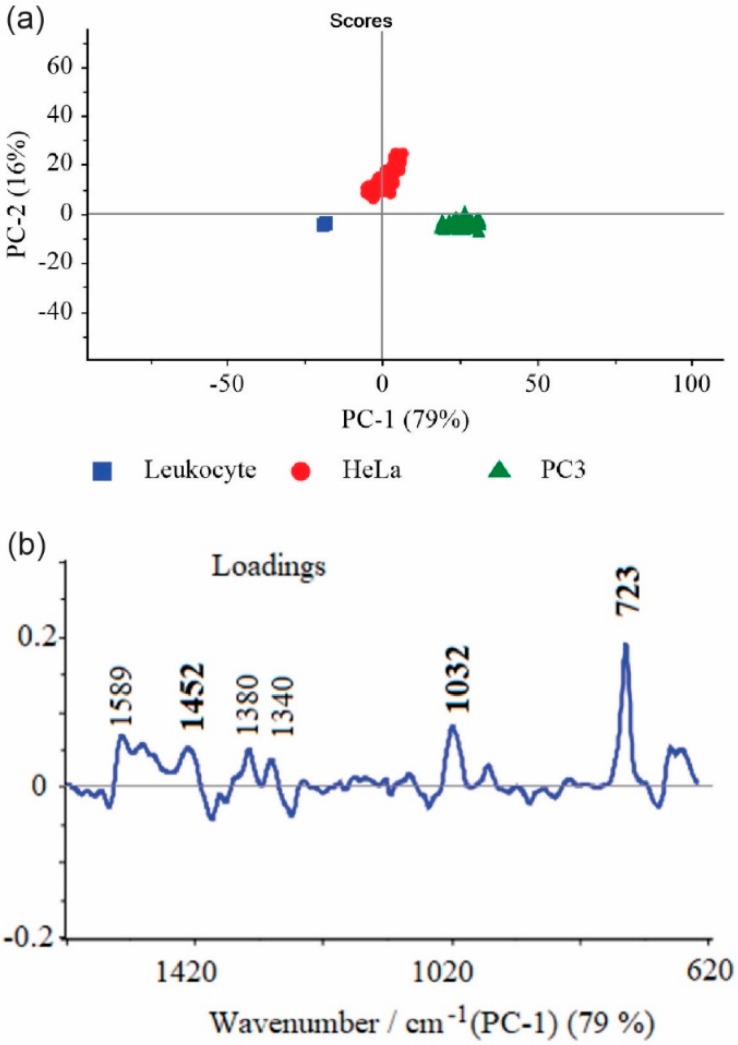
(**a**) The score plots of PC-1 versus PC-2 components for differentiation of leucocyte, HeLa, and PC3 cells. PCA was calculated for the whole region (600–1700 cm^−1^); (**b**) PC-1 loading plot.

The principal component analysis (PCA) was also performed for the statistical analysis of all studied cells. The data sets including 300 spectra obtained from HeLa, PC3, and leucocyte were analyzed by PCA using the commercial Unscrambler® software (CAMO software AS, version 10.3, Oslo, Norway). It is obvious from looking at Figure 6 that the SERS spectra of different cell types could be easily distinguished (all SERS data were divided into three clusters corresponding to leucocytes (blue), PC3 (green), and HeLa (red) based on the significant PCs (PC-1, PC-2)). We could clearly see that they were distributed into separated regions, which indicated the possibility of differentiation of analyzed cells. The PC-1 and PC-2 were counted in the wavenumber region between 600 and 1700 cm^−1^ and differentiated the cancer cells from normal cells with a sensitivity of 82%.

It should be noticed that the cluster of leucocytes showed a relatively higher homogeneity in comparison to HeLa and PC3 cells, which probably reflects the molecular changes in the structure and biochemical composition of cancer cells [58].

As it can be seen from Figure 4 and Figure 6b many spectral features present in SERS spectra were captured by the main PCs.

The prominent SERS bands at *ca.* 725, 1032, and 1452 cm^−1^ also had the largest weights in the variations and indicated the most significant differences among the three types of analyzed cells. The important contribution to PC-1 also gave bands at *ca.* 1589, 1553, 1470, 1368, and 1380 cm^−1^, analyzed in the previous section. To enhance the sensitivity of differentiation among analyzed cells, a further PCA calculation was made on the limited SERS data (the region between 700 and 750 cm^−1^) where one of the most prominent marker bands at 725 cm^−1^ was observed (Figure S3). In this case, the calculated PC-1 and PC-2 values increased and explained up to 98% of the total variance. This result illustrated that all studied cells were clearly separated into three clusters corresponding to the prostate cancer (PC3), cervical carcinoma (HeLa), and leucocyte cell lines, respectively (Figure S3A).

## 5. Conclusions

Surface-enhanced Raman spectroscopy can be used as an ultrasensitive (at the level of single cell), non-invasive, rapid, and label-free method that provides valuable structural and biochemical analysis of circulating tumor cells. Moreover, Raman spectroscopy coupled with multivariate techniques gives the statistical diagnostic approach for efficient screening of cancer cells.

Our work clearly demonstrates the potential of such a PCA-based SERS method for the direct detection and identification of prostate cancer (PC3) and cervical carcinoma (HeLa) cell lines in blood samples with excellent specificity and sensitivity. We offer a single technology that is optimal for: (i) isolation of CTCs from blood samples, and their (ii) enrichment, (iii) detection, and (iv) molecular analysis.

The developed strategy is based on the tailor-made membrane-based SERS platforms, prepared according to a novel procedure, which permits at that same time the separation, immobilization/enrichment, and enhancement of the week Raman signals of CTCs.

The diagnostic sensitivity of 98% can be achieved for differentiation of PC3, HeLa, and normal cells.

The presented SERS-based strategy for circulating cell detection offers, besides such unique advantages as an ability for rapid and label-free recognition of CTCs with excellent sensitivity and selectivity, also simple sample preparation and cost-effective measurements. Our results indicate that a SERS-based cancer sensor has a great potential to be introduced in a variety of studies conducted on different types of cancer cells, especially from clinical samples.

## Figures and Tables

**Table 1:** Assignment of SERS bands depicted in Figure 4 [49,53,54,55,56,57].

Observed SERS Band (cm^−1^)	Protein	Lipids	Nucleic Acid
652–658	Tyr (C–C twist)		
725–730	Trp	C–N head group choline (H_3_C)_3_N+	A
785			PO_2_ symm
827			RNA backbone
850	Tyr, Pro		
890	Structural protein modes of tumors		
925	C–C str alpha-helix, Pro, Val		
960	CH_3_ def	CH_3_ def	
1003	Phe		
1030–1032		CH_2_CH_3_ bending modes of lipids	
1093	C–N stretch	CC str chain, C–O str	PO_2_ symm
1128	C–N str bk	porphyrin	
1170–1172	Tyr C–H inplane		T
1210	C–C_6_H_5_ str in phenylalanine tyrosine		
1243	Amide III (beta sheet)		
1267–1270	Amide III ( random coil)	=CH def	
1319	CH_3_ def, collagen	CH_3_CH_2_ twist	G
1324			purine bases of DNA
1345			A, G
1370		sphingoglycolipids	
1452	structural protein modes of tumors		
1458			A, G
1552			A, G
1597–1600	Phe, Tyr		
1616–1618	C≡C str of Tyr and Trp		
1657–1665	Amide I	C=C str	

## Supplementary Materials

The following are available online at https://www.mdpi.com/2079-4991/9/3/366/s1, Figure S1: Reference SERS spectra of (a) HeLa, (b) PC3, and (c) leucocytes placed directly on SERS substrate, Figure S2: SERS spectra of (a) leucocytes, (b) HeLa, and (c) PC3 cells recorded from different spots (ca. 100) within the same sample. The excitation wavelength was at 785 nm, laser power was 5 mW, and the acquisition time was 60 seconds, Figure S3: (a) First three PC (PC-1, PC-2, and PC-3) scores plot of 3D-PCA calculated for narrow range (700–750 cm^−1^) and (b) loadings plot of the first principal component showing the most prominent marker band at 723 cm^−1^,Table S1: The sizes of blood components, based on data from Handin et al., Table S2: The RSD of the selected intensities of SERS signals of leucocytes, HeLa, and PC3 cells recorded from 1000 different spots within the same sample.
