# Supplementary material for: Detection of Circulating Tumor Cells Using Membrane-Based SERS Platform: A New Diagnostic Approach for ‘Liquid Biopsy’
Source: Nanomaterials (Basel). 2019 Mar 5;9(3):366. doi: 10.3390/nano9030366 (PMC6473992; doi:10.3390/nano9030366)
Supplement: Supplementary file 1 [file nanomaterials-09-00366-s001.pdf]

Supplementary Materials

Detection of Circulating Tumor Cells Using Membrane-Based SERS Platform: A New Diagnostic Approach for 'Liquid Biopsy'

Agnieszka Kamińska [1,*], Tomasz Szymborski [1], Evelin Witkowska [1], Ewa Kijeńska-Gawrońska [2], Wojciech Świeszkowski [2], Krzysztof Niciński [1], Joanna Trzcińska-Danielewicz [3] and Agnieszka Girstun [3]

[1] Institute of Physical Chemistry, Polish Academy of Sciences, Kasprzaka 44/52, 01-224 Warsaw, Poland; tszymborski@gmail.com (T.S.); ewitkowska@ichf.edu.pl (E.W.); knicinski@ichf.edu.pl (K.N.)
[2] Faculty of Materials Science and Engineering, Warsaw University of Technology, Wołoska 141, 02-507 Warsaw, Poland; ewakijenska@gmail.com (E.K.-G.); wojciech.swieszkowski@pw.edu.pl (W.S.)
[3] Department of Molecular Biology, Institute of Biochemistry, Faculty of Biology, University of Warsaw, Miecznikowa 1, 02-096 Warsaw, Poland; jtd@biol.uw.edu.pl (J.T.-D.); agirstun@biol.uw.edu.pl (A.G.)
* Correspondence: akamin@ichf.edu.pl

1. Components of Blood

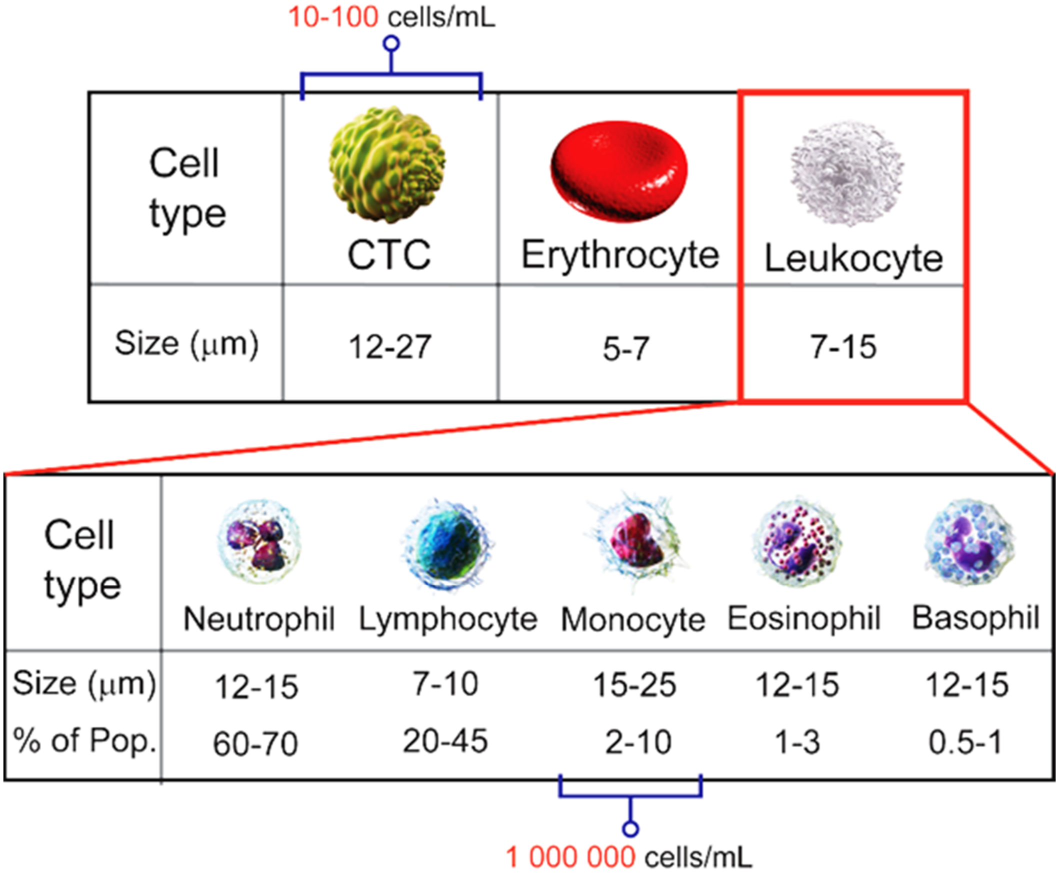

Cell type	CTC	Erythrocyte	Leukocyte
Size (μm)	12-27	5-7	7-15

Cell type	Neutrophil	Lymphocyte	Monocyte	Eosinophil	Basophil
Size (μm)	12-15	7-10	15-25	12-15	12-15
% of Pop.	60-70	20-45	2-10	1-3	0.5-1

Table S1. The sizes of blood components, based on data from Handin et al. [1]

2. Reference SERS spectra

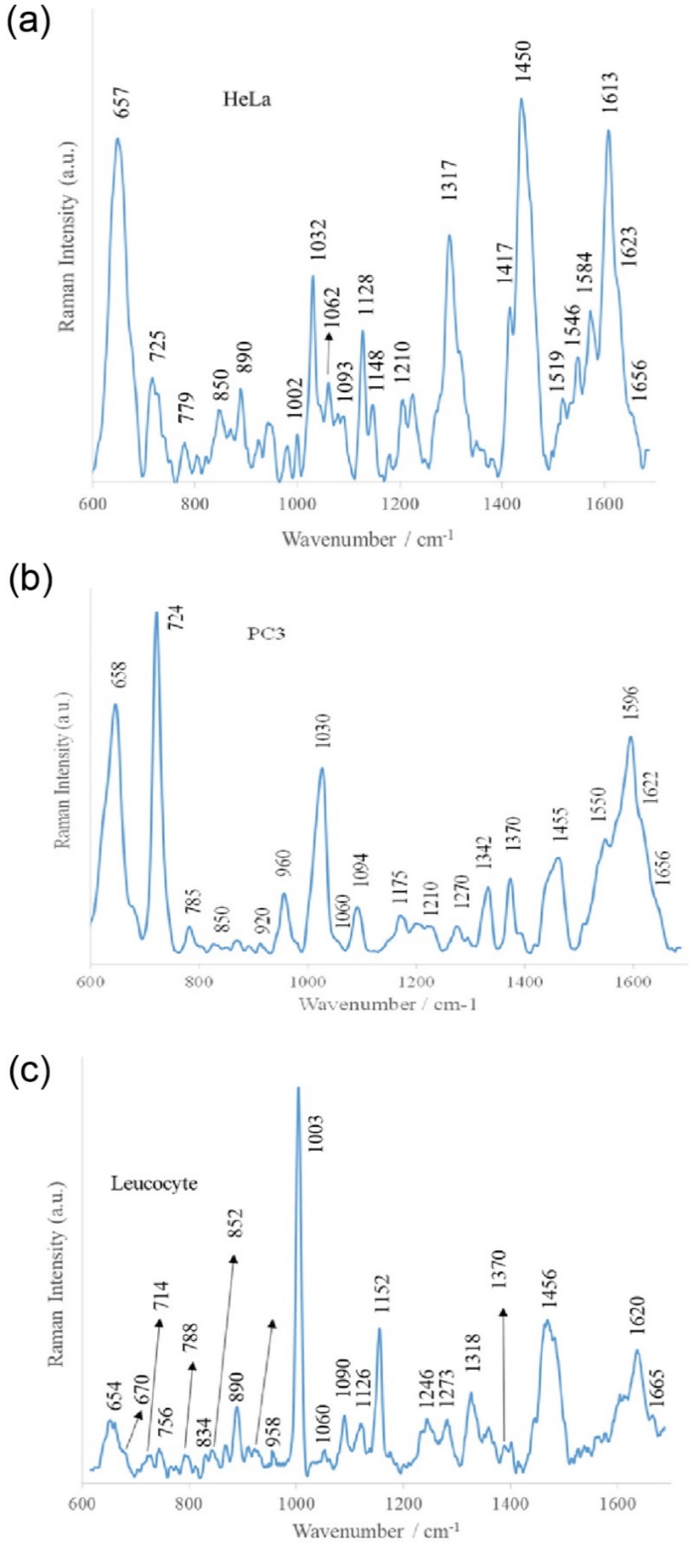

Figure S1. Reference SERS spectra of **(a)** HeLa, **(b)** PC3, and **(c)** leucocytes placed directly on SERS substrate.

3. Reproducibility of measured spectra

The reproducibility of the measured SERS signals plays a crucial role in the analytical and biomedical applications of SERS technique. The average standard deviation (Av. STD) of the SERS signals of leucocytes, HeLa, and PC3 cells were calculated and presented in the Table S2. The SERS spectra of these cells recorded from different spots within the same samples were also presented in Figure S2.

Table S2. The RSD of the selected intensities of SERS signals of leucocytes, HeLa, and PC3 cells recorded from 1000 different spots within the same sample.

Cell type	Selected bands [cm^{-1}]	RSD (%)
Leucocytes	1003	8
HeLa	1035	7.5
PC3	725	6.2

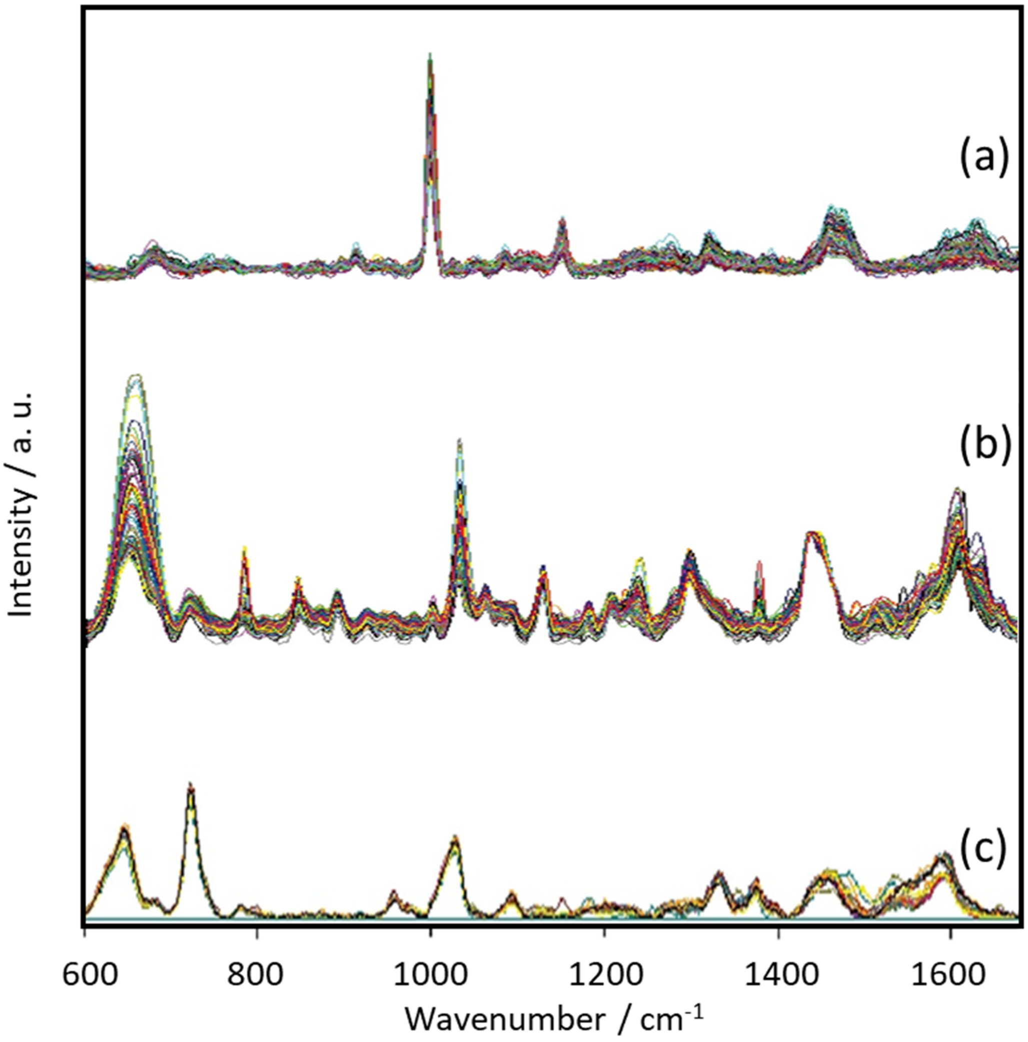

Figure S2. SERS spectra of **(a)** leucocytes, **(b)** HeLa, and **(c)** PC3 cells recorded from different spots (ca. 100) within the same sample. The excitation wavelength was at 785 nm, laser power was 5 mW, and the acquisition time was 60 seconds.

3. Principal Component Analysis

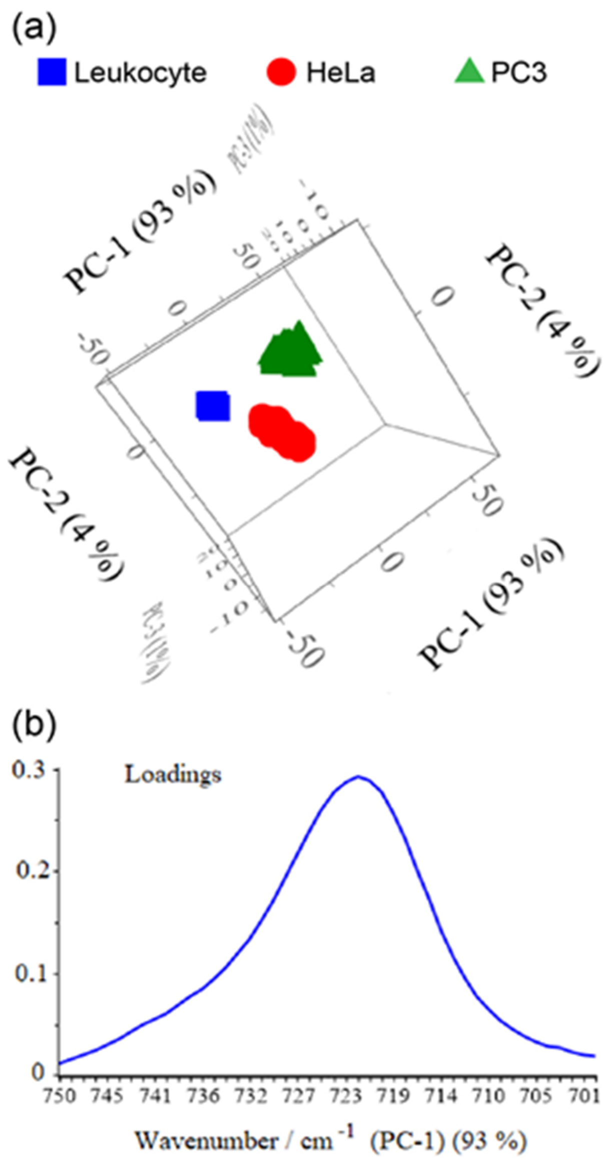

Figure S3. (a) First three PC (PC-1, PC-2, and PC-3) scores plot of 3D-PCA calculated for narrow range (700-750 cm^{-1}) and **(b)** loadings plot of the first principal component showing the most prominent marker band at 723 cm^{-1}.

References:

[1] R. I. Handin, S. E. Lux and T. P. Stossel, *Blood: Principles and Practice of Hematology*, Lippincott Williams & Wilkins, Philadelphia, 2004.
